# AI-Quantitative CT Coronary Plaque Features Associate With a Higher Relative Risk in Women: CONFIRM2 Registry

**DOI:** 10.1161/CIRCIMAGING.125.018235

**Published:** 2025-03-31

**Authors:** Gudrun M. Feuchtner, Pietro G. Lacaita, Jeroen J. Bax, Fatima Rodriguez, Rine Nakanishi, Gianluca Pontone, Saima Mushtaq, Ronny R. Buechel, Christoph Gräni, Amit R. Patel, Cristiane C. Singulane, Andrew D. Choi, Mouaz Al-Mallah, Daniele Andreini, Ronald P. Karlsberg, Geoffrey Cho, Carlos E. Rochitte, Mirvat Alasnag, Ashraf Hamdan, Filippo Cademartiri, Erica Maffei, Hugo Marques, Pedro M. Gonçalves Pereira, Himanshu Gupta, Martin Hadamitzky, Omar Khalique, Dinesh Kalra, James D. Mills, Nick S. Nurmohamed, Paul Knaapen, Matthew Budoff, Kashif Shaikh, Enrico Martin, David M. German, Maros Ferencik, Andrew C. Oehler, Roderick Deaño, Prashant Nagpal, Marly Van Assen, Carlo Nicola De Cecco, Borek Foldyna, Jan Michael Brendel, Victor Y. Cheng, Kelley Branch, Marcio Bittencourt, Sabha Bhatti, Venkateshwar Polsani, George Wesbey, Rhanderson Cardoso, Ron Blankstein, Augustin Delago, Amit Pursnani, Amro Alsaid, Stephen Bloom, Vasileios Kamperidis, Fabian Barbieri, Melissa Aquino, Ibrahim Danad, Alexander van Rosendael

**Affiliations:** 1 Department of Radiology, Medical University of Innsbruck, Austria (G.M.F., P.G.L.).; 2 Department of Cardiology, Leiden University Medical Center, the Netherlands (J.J.B., A.R.).; 3 Division of Cardiovascular Medicine, Stanford University School of Medicine, Paolo Alto, CA (F.R.).; 4 Department of Cardiovascular Medicine, Toho University Graduate School of Medicine, Tokyo, Japan (R.N.).; 5 Department of Perioperative Cardiology and Cardiovascular Imaging, Centro Cardiologico Monzino Istituto di Ricerca e Cura a Carattere Scientifico (IRCCS), Milan, Italy (G.P.).; 6 Department of Biomedical, Surgical and Dental Sciences, University of Milan, Italy (G.P.).; 7 Perioperative Cardiology and Cardiovascular Imaging Department, Centro Cardiologico Monzino IRCCS, Milan, Italy (S.M.).; 8 Department of Nuclear Medicine, Cardiac Imaging, University Hospital and University of Zurich, Switzerland (R.R.B.).; 9 Department of Cardiology, Inselspital, Bern University Hospital, University of Bern, Switzerland (C.G.).; 10 Division of Cardiovascular Medicine, University of Virginia, Charlottesville (A.R.P., C.C.S.).; 11 Department of Cardiology and Radiology, George Washington University, Washington, DC (A.D.C., ).; 12 UNICA, Unit of Cardiovascular Imaging, Hospital da Luz, Lisboa, Portugal (H.M., P.M.G.F); 13 Department of Cardiology, Houston Methodist, TX (M.A.-M.,.).; 14 Division of University Cardiology, IRCCS Galeazzi Sant’Ambrogio, Department of Biomedical and Clinical Sciences, University of Milan, Italy (D.A.); 15 Cardiovascular Research Foundation of Southern California, Cedars-Sinai Heart, David Geffen School of Medicine, University of California Los Angeles (R.P.K., G.C.).; 16 Heart Institute, InCor, University of São Paulo Medical School, Brazil (C.E.R.).; 17 Cardiac Center, King Fahad Armed Forces Hospital, Jeddah, Saudi Arabia (M. Alasnag).; 18 Department of Cardiology, Rabin Medical Center, Petah Tikva and Tel Aviv University, Israel (A.H.).; 19 IRCCS SYNLAB SDN, Naples, Italy (F.C.).; 20 Department of Radiology, Istituto di Ricovero e Cura a Carattere (IRCCS) SYNLAB SDN, Naples, Italy (E. Maffei).; 21 Cardiac Imaging, Heart and Vascular Institute, Valley Health System, Paramus, NJ (H.G.).; 22 Department of Radiology and Nuclear Medicine, German Heart Center Munich, Germany (M.H.).; 23 Division of Cardiovascular Imaging, St. Francis Hospital and Heart Center, Roslyn, NY (O.K.).; 24 Division of Cardiology, Department of Medicine, University of Louisville School of Medicine, KY (D.K.).; 25 Department of Cardiology, West Virginia University Heart and Vascular Institute, Morgantown (J.D.M.).; 26 Department of Cardiology and Department of Vascular Medicine, Amsterdam UMC, Vrije Universiteit Amsterdam, the Netherlands (N.S.N.).; 27 Department of Cardiology, Amsterdam UMC, Vrije Universiteit Amsterdam, the Netherlands (P.K.).; 28 The Lundquist Institute, Torrance, CA (M. Budoff).; 29 University of Tennessee Medical Center, Knoxville (K.S.).; 30 Division of Cardiology, MercyOne-Iowa Heart Center, Des Moines, IA (E. Martin).; 31 Knight Cardiovascular Institute, Oregon Health & Science University, Portland (D.M.G., M.F.).; 32 Allegheny Health Network Cardiovascular Institute, Allegheny Health Network, Pittsburgh, PA (A.C.O.).; 33 Division of Cardiovascular Medicine, Department of Medicine, University of Wisconsin School of Medicine and Public Health, Madison (R.D.).; 34 Department of Radiology, University of Wisconsin School of Medicine and Public Health, Madison (P.N.).; 35 Department of Radiology and Imaging Sciences, Emory University, Atlanta, GA (M.V.A., C.N.D.C.).; 36 Department of Radiology, Cardiovascular Imaging and Research Center, Massachusetts General Hospital and Harvard Medical School, Boston (B.F., J.M.B.).; 37 Minneapolis Heart Institute, MN (V.Y.C.).; 38 Division of Cardiology, Cardiovascular Clinical Trials, University of Washington, Seattle (K.B.).; 39 Heart and Vascular Institute, University of Pittsburgh Medical Center, PA (M. Bittencourt).; 40 National Institute of Cardiovascular Diseases, Karachi, Pakistan (S. Bhatti).; 41 Piedmont Heart Institute, Atlanta, GA (V.P.).; 42 Department of Cardiovascular Computed Tomography, Scripps Clinic, La Jolla, CA (G.W.).; 43 Division of Cardiovascular Medicine, Brigham and Women’s Hospital, Boston, MA (R.C., R.B.).; 44 Medical Data Research Collaborative, United Kingdom (A.D.).; 45 Department of Medicine, Mount Auburn Hospital, Harvard Medical School, Cambridge, MA (A.D.).; 46 Cardiology, Endeavor NorthShore Cardiovascular Institute, Evanston, IL (A.P.).; 47 Pritzker School of Medicine, University of Chicago, IL (A.P.).; 48 Department of Cardiac Imaging, Baylor Scott & White The Heart Hospital Plano, TX (A.A.).; 49 Midwest Heart and Vascular Associates, Kansas City, MO (S. Bloom).; 50 1st Cardiology Department, Medical School, Aristotle University of Thessaloniki, Greece (V.K.).; 51 Department of Cardiology, Angiology and Intensive Care Medicine, Deutsches Herzzentrum der Charité, Berlin, Germany (F.B.).; 52 Cleerly Health, Denver, CO (M. Aquino).; 53 Department of Cardiology, Radboud University Medical Center, Nijmegen, the Netherlands (I.D.).

**Keywords:** artificial intelligence, atherosclerosis, computed tomography, computed tomography angiography, coronary artery disease, women’s health

## Abstract

**BACKGROUND:**

Coronary plaque features are imaging biomarkers of cardiovascular risk, but less is known about sex-specific patterns in their prognostic value. This study aimed to define sex differences in the coronary atherosclerotic phenotypes assessed by artificial intelligence–based quantitative computed tomography (AI-QCT) and the associated risk of major adverse cardiovascular events (MACEs).

**METHODS:**

Global multicenter registry including symptomatic patients with suspicion of coronary artery disease referred for coronary computed tomography angiography. AI-QCT analyzed 16 coronary artery disease features. The primary end point was MACE defined as death, myocardial infarction, late revascularization, cerebrovascular events, unstable angina, and congestive heart failure.

**RESULTS:**

Among 3551 patients (mean age, 59±12 years; 49.5% women), MACE occurred in 3.2% of women and 6.1% of men during an average follow-up of 4.8±2.2 years. The AI-QCT features total plaque volume, noncalcified plaque, calcified plaque, and percentage atheroma volume were significantly higher in men (*P*<0.001), and high-risk plaques were more prevalent (9.2% versus 2.5%; *P*<0.0001). Independent of age and cardiovascular risk factors, the AI-QCT-derived features of total plaque volume, noncalcified plaque, calcified plaque, and percentage atheroma volume conferred a higher relative risk of MACE in women than men. For every 50-mm^3^ increase in total plaque volume, relative risk increased by 17.7% (95% CI, 1.12–1.24) in women versus 5.3% (95% CI, 1.03–1.07) in men (*P*_interaction_<0.001); for noncalcified plaque, relative risk increased by 27.1% (95% CI, 1.17–1.38) versus 11.6% (95% CI, 1.08–1.15; *P*_interaction_=0.0015); and for calcified plaque, relative risk increased by 22.9% (95% CI, 1.14–1.33) versus 5.4% (95% CI, 1.01–1.10; *P*_interaction_=0.0012), respectively. Similarly, for percentage atheroma volume, the risk was higher in women. The findings remained unchanged when restricted to a secondary composite end point (death and myocardial infarction).

**CONCLUSIONS:**

The AI-QCT plaque features, total plaque volume, noncalcified plaque, calcified plaque, and percentage atheroma volume, conferred a higher relative MACE risk in women and may prompt more aggressive antiatherosclerotic therapy and reinforced preventive interventions.

**REGISTRATION:**

URL: https://www.clinicaltrials.gov; Unique identifier: NCT04279496.

**Clinical Perspective**

The artificial intelligence–based quantitative computed tomography plaque features, total plaque volume, noncalcified plaque, calcified plaque, and percentage atheroma volume, conferred a higher relative major adverse cardiovascular event risk in women and may prompt more aggressive antiatherosclerotic therapy and reinforced preventive interventions in women with high plaque burden. Artificial intelligence–based quantitative computed tomography plaque features should be integrated into existing risk scores to enhance their accuracy. Risk stratification based on artificial intelligence–based quantitative computed tomography may need to be sex-specific.

**See Editorial by Shaw and Lin**

Coronary artery disease (CAD) is the leading cause of death among women globally, affecting over 60 million women in the United States alone.^1^ Remarkably, CAD is responsible for more deaths in women than in men (43% versus 38%).^1^ Women are currently underdiagnosed and undertreated for CAD.^2,3^ Therefore, early identification of women at risk and timely implementation of preventive strategies hold the potential to reduce CAD-related mortality and morbidity. However, CAD exhibits profound sex-based differences in clinical presentation,^4^ incidence, and outcomes,^2^ underscoring the need for a tailored approach to diagnosis and management. In women, CAD generally manifests approximately a decade later than in men due to the protective effects of estrogen.^5^ Despite this delayed onset, women with CAD frequently experience worse outcomes,^2^ which are particularly worse among young women (<55 years).^6^ Furthermore, women more often present with diffuse, nonobstructive disease^2^ and have different coronary artery calcium patterns^7^ and lower low-attenuation plaque volumes,^8^ reflecting sex-specific vulnerabilities that warrant further investigations.

An important diagnostic imaging strategy in patients with suspected CAD is coronary computed tomography angiography (CCTA),^9^ which provides high-resolution imaging of the coronary vasculature, enabling detailed assessment of atherosclerosis. Novel artificial intelligence–based quantitative computed tomography (AI-QCT)^10–12^ allows for an even more nuanced noninvasive plaque characteristic analysis by enabling an automated quantification of total plaque volumes (TPVs),^10^ noncalcified plaque (NCP) and calcified plaque (CP) volumes, and high-risk plaque (HRP) features.^13,14^ Thus, AI-QCT offers the potential to enhance diagnostic precision, with the potential for refining risk prediction and stratification in women, with commercially available technology.

Therefore, in this study, we aimed to define sex differences in CAD features using AI-QCT for predicting major adverse cardiovascular events (MACEs) in symptomatic patients without prior CAD, utilizing data from the Quantitative Coronary Computed Tomography Angiography Evaluation for Evaluation of Clinical Outcomes: an International, Multicenter Registry.

## Methods

### Study Design and Population

The Quantitative Coronary Computed Tomography Angiography Evaluation for Evaluation of Clinical Outcomes: an International, Multicenter Registry is an ongoing multicenter, international, observational cohort study, in which details of the study design have been published previously.^15^ Participating sites retrospectively included sequential patients with a clinically indicated CCTA of ≥64-detector rows. Exclusion criteria for enrollment were age <18 years, absence of CCTA data, or follow-up information for clinical events, pregnancy, or a noncardiac illness with life expectancy <2 years. For this analysis, we included symptomatic patients (defined as either atypical or typical chest pain, nonspecific chest pain, dyspnea, palpitations, syncope, or other nonspecific symptoms possibly of cardiac origin), without a prior history of CAD (defined as a prior myocardial infarction [MI], prior percutaneous coronary intervention, coronary artery bypass grafting, or known ≥50% stenosis on invasive coronary angiography), who had at least 3-year follow-up for clinical events and who underwent CCTA for the evaluation of CAD. The study was approved by the institutional review board of each participating site, and subjects gave written informed consent (unless waived by each individual institutional review board due to the retrospective study design).

### Data Availability Statement

Data will not be shared publicly.

### Study End Points

Primary end point was MACE, defined by the occurrence of any of the following events: all-cause death; myocardial Infarction, defined in accordance with the Universal Definition of Myocardial Infarction^16^; cerebrovascular accident, defined as a neurological deficit lasting ≥24 hours or lasting <24 hours with a brain imaging study showing infarction; late coronary revascularization, defined as revascularization ≥90 days following the index diagnostic test; and unstable angina, defined as hospitalization for signs or symptoms of unstable angina. Unstable angina was defined as (1) rest angina, (2) new-onset angina (<2 months), or (3) increasing angina (in intensity, duration, and frequency) and congestive heart failure according to the Framingham Heart Study criteria. All events were adjudicated by a Clinical Events Committee and verified by clinical reports from the individual sites. Secondary end point was death and MI. Time to event was defined as the duration from the baseline CCTA to an event or last follow-up; 0% of patients were censored at 2.75 years at both baseline CCTA and 8% before 3 years. For analysis purposes, follow-up data are truncated at 3 years because this was the minimum required follow-up.

### AI-QCT Analysis

Artificial intelligence–enabled technology (Cleerly, Inc, Denver) was applied to analyze the CCTA images.^17^ This US Food and Drug Administration–cleared software service utilizes multiple validated convolutional neural networks for image quality assurance, coronary artery vessel segmentation and labeling, luminal wall and vessel contour definition, and plaque volume quantification and characterization based on their computed tomography attenuation (Hounsfield Units [HU]). Prior validation of this AI-QCT technology has been conducted.^18–21^ The algorithm selects the series with the best image quality for CCTA analysis on a per-vessel basis. Finally, a trained radiological technologist provides a quality assurance overview of the artificial intelligence analysis.

Coronary segments with a diameter ≥1.5 mm were included using the modified 18-segment Society of Cardiovascular Computed Tomography model.^22^ Coronary percentage stenosis was classified on a per-vessel basis as per Society of Cardiovascular Computed Tomography guidelines and categorized by the CAD Reporting and Data System.^23^

### AI-QCT Coronary Plaque Features

Each coronary segment was evaluated for the presence of plaque, defined as any tissue structure >1 mm^2^ within the coronary artery wall that was differentiated from the surrounding epicardial fat or the vessel lumen itself. TPV (mm^3^) was defined as the sum of all plaque volumes calculated for each coronary lesion among all vessels (left main, left anterior descending, circumflex, right coronary artery, and side branches). Noncalcified plaque (NCP) was defined as lesion with a computed tomography density between 30 and +350 HU and CP above >350 HU.^24^ Low-attenuation plaque was defined as a plaque with a computed tomography density <30 HU and a volume of >2 mm^3^.^13^ Percentage atheroma volume (PAV) was normalized to the total per-patient vessel volume to account for the variation in coronary artery volume and calculated as plaque volume/vessel volume×100%.^25^ Positive arterial remodeling was calculated by examining the lesion diameter divided by the normal reference diameter and defined as a ratio ≥1.1.^26^ HRP was defined as defined as coronary lesions with the presence of both 2 features: low-attenuation plaque <30 HU and positive remodeling. When poor image quality was present due to motion artifacts, low contrast intravascular attenuation, beam hardening, or other artifacts, only the part of the coronary artery with poor quality was excluded from the analysis.

### AI-QCT Extent of Vessel Disease

Because a high number of patients have small volumes of atherosclerosis, nonobstructive disease was defined as >16 mm^3^ of plaque volume. None/minimal disease was defined as ≤16 mm^3^ of plaque volume.^10^ One, 2, and 3 vessels or left main disease were differentiated. Obstructive disease was defined as >50% stenosis.

#### Clinical Data Collection

Cardiovascular risk factors (CVRFs) were collected according to standardized definitions as described in the study protocol^15^ and retrieved from the electronic medical record baseline before the CCTA. Systemic arterial hypertension was defined as a blood pressure of ≥140/90 mm Hg, a documented history of hypertension, or current treatment with antihypertensive medications. Diabetes was defined by a prior physician diagnosis and the use of insulin or oral hypoglycemic agents. Dyslipidemia was defined as either untreated dyslipidemia or current lipid-lowering medication. Smoking history was classified as positive if the patient was a current smoker or had quit smoking within 3 months. Family history of CAD was based on patient self-report. Symptom presentations were categorized as follows: typical chest pain, atypical chest pain, noncardiac pain, dyspnea, palpitations, syncope, other, or asymptomatic. The Diamond and Forrester (DF) pretest probability was calculated,^27^ and the atherosclerotic cardiovascular disease risk (ASCVD) score (2013).^28^ Data management including documentation was performed by e-case report form through a secure Web portal (RedCap), with consistency and validation checks within the electronic data capture system. Primary data collection based on source-documented hospital and clinic chart reviews was performed.

#### Statistical Analysis

It was performed with SAS 9.4 (SAS Institute, Inc, Cary, NC). Continuous variables were assessed for normality by the Shapiro-Wilk test. Normally distributed variables are presented as mean±SD, and nonnormally distributed variables are presented as median and interquartile range. Categorical variables are expressed as absolute numbers and percentages.

Univariate regression analysis was performed to evaluate potential predictors of MACE for all coronary AI-QCT features, and demographic and baseline clinical variables, such as CVRF and comorbidities, split for men and women. Multivariate Cox proportional hazard regression models were calculated and adjusted for age and the CVRFs, for all AI-QCT features (TPV, NCP, CP, and PAV) separately.

Receiver operating characteristic curves were generated for CVRFs, DF, and AI-QCT parameters and combined models (CVRF+AI-QCT features) to evaluate their discriminatory ability in predicting clinical outcomes, including the use of the c-statistic, in both men and women, and sex differences were tested. The same receiver operating characteristic curve models were repeated for DF instead of CVRF.

For the evaluation of the absolute differences in risk of MACE at 3 years based on the level of NCP volume, a model was fit using logistic regression. The model included covariates for NCP, sex, and the interaction of the 2. The results are plotted as 2 lines with 95% CI showing the resulting predicted probabilities of MACE for men and women by NCP volumes.

## Results

### Demographics

This analysis included 4163 symptomatic patients from 18 centers across 11 countries, recruited between 2018 and 2022. After excluding 288 asymptomatic patients, 322 patients with a prior CAD history, and 2 patients with incomplete clinical information, the final cohort comprised 3551 patients of which 49.5% were women (Table 1). The majority of women (77.7%) and men (80.5%) were White. Body mass index and most major CVRF were comparable between the sexes. Women presented more often with atypical chest pain (39.5% versus 35.7%; *P*=0.019), while other symptoms were comparable between sexes. There were no significant sex differences in rates of heart failure, atrial fibrillation, or peripheral arterial disease, and the overall prevalence was low (<10%). Baseline medication usage was comparable between sexes (Table 1).

**Table 1.**
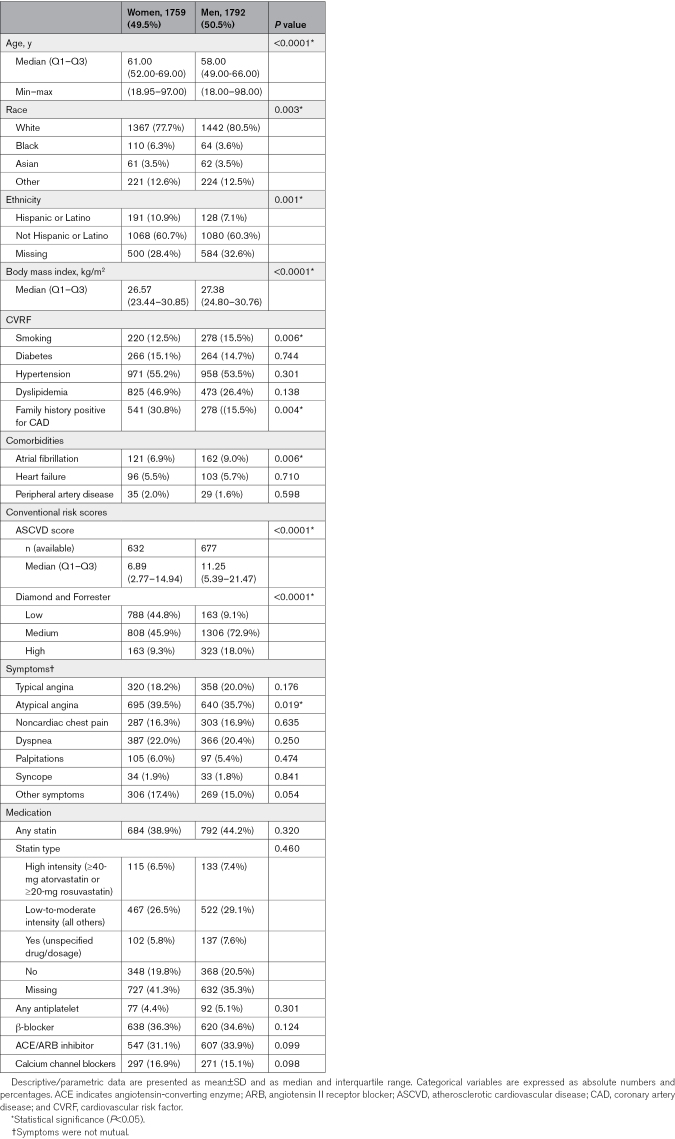
Demographics/Risk Factors

### Quantitative Coronary Atherosclerosis Evaluation by AI-QCT

In general, all AI-QCT plaque features were lower or less prevalent in women (Table 2), concerning TPV, NCP, CP, PAV, low-attenuation plaque, HRP, diameter stenosis (DS), and extent of vessel disease (*P*<0.001 between sexes for all variables). The volumes of TPV, NCP, and CP were by both mean and median approximately half in women compared with men. Specifically, the rate of none/minimal CAD was higher in women than in men (31% versus 19.1%; *P*<0.0001), and the prevalence of HRP was lower in women (2.5% versus 9.2%; *P*<0.0001). Obstructive CAD (≥50% stenosis) was present in 8.8% of women and 20.2% of men (*P*<0.0001).

**Table 2.**
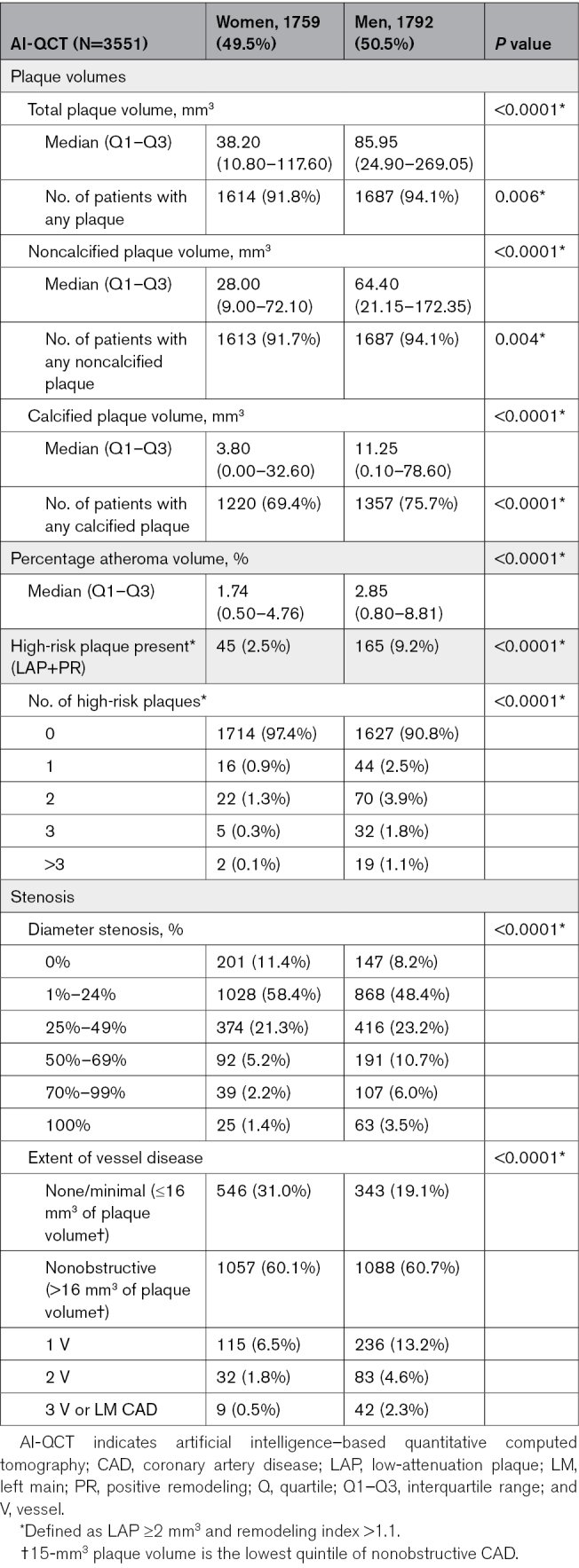
Quantitative AI-QCT Coronary Artery Disease Characteristics

### Primary End Point: MACEs

During a mean follow-up duration of 4.8±2.2 years, the overall MACE rate was 167 (4.8%) per patient, with a lower rate in women (3.2%) than men (6.1%; *P*<0.001). There were 34 deaths, 24 MIs, 12 cerebrovascular accidents, 23 hospitalizations for congestive heart failure, 17 unstable angina events, and 84 late revascularizations. There was no significant interaction between age and sex in predicting MACE (*P*_interaction_=0.846).

### Association of Quantitative Coronary Atherosclerotic Plaque Characteristics as Assessed by AI-QCT With MACE

Sex (men versus women) was associated with an increased risk of MACE (hazard ratio [HR], 1.92 [95% CI, 1.38–2.64]; *P*<0.0001) on univariate regression. All AI-QCT features were associated with MACE in both men and women, with generally higher relative risk observed in women (Table 3). The HR for 50-mm^3^ increase in plaque was significantly higher in women versus men for TPV of 1.19 (95% CI, 1.14–1.23) versus 1.06 (95% CI, 1.04–1.07; *P*_interaction_<0.0001), NCP of 1.35 (95% CI, 1.26–1.45) versus 1.12 (95% CI, 1.08–1.15; *P*_interaction_<0.0001), and CP of 1.24 (95% CI, 1.17–1.31) versus 1.07 (95% CI, 1.04–1.11; *P*_interaction_<0.0001). Also, the relative risk associated with HRP was greater in women: HR, 5.77 versus 2.13 (*P*_interaction_=0.0363). When plaque is adjusted for vessel volume as done by PAV, similar findings were observed: HR, 1.65 (95% CI, 1.46–1.86) in women versus 1.31 (95% CI, 1.22–1.41) in men (*P*_interaction_=0.0012). Women within the highest TPV stage (>750-mm^3^ plaque volume) were at a higher relative risk than men: HR, 21.8 (95% CI, 9.62–49.41) versus 4.41 (95% CI, 2.46–7.88; *P*_interaction_=0.0018).

**Table 3.**
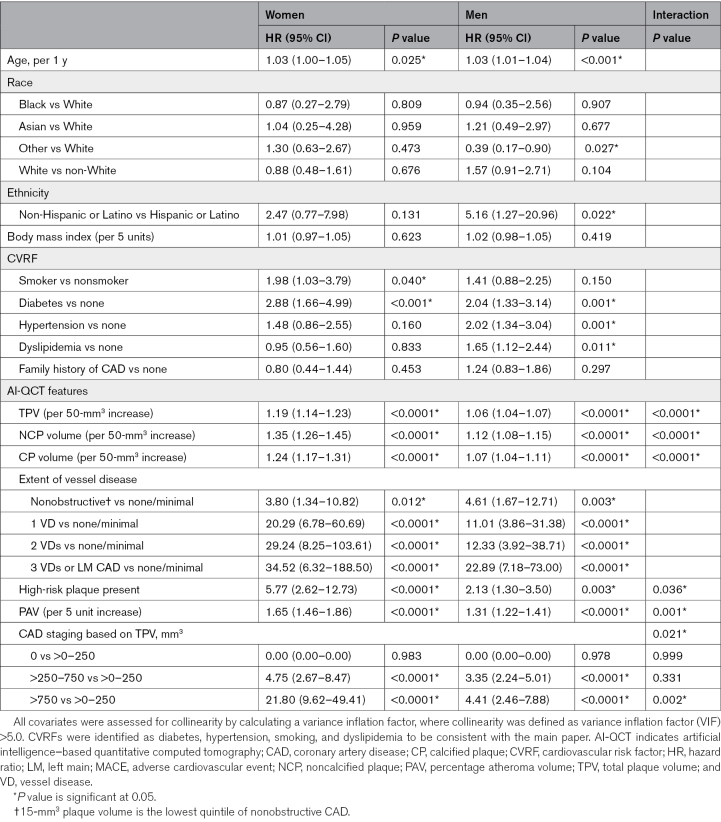
Univariate Analysis: CVRFs and AI-QCT Features: Prediction of MACE

**Figure 1.**
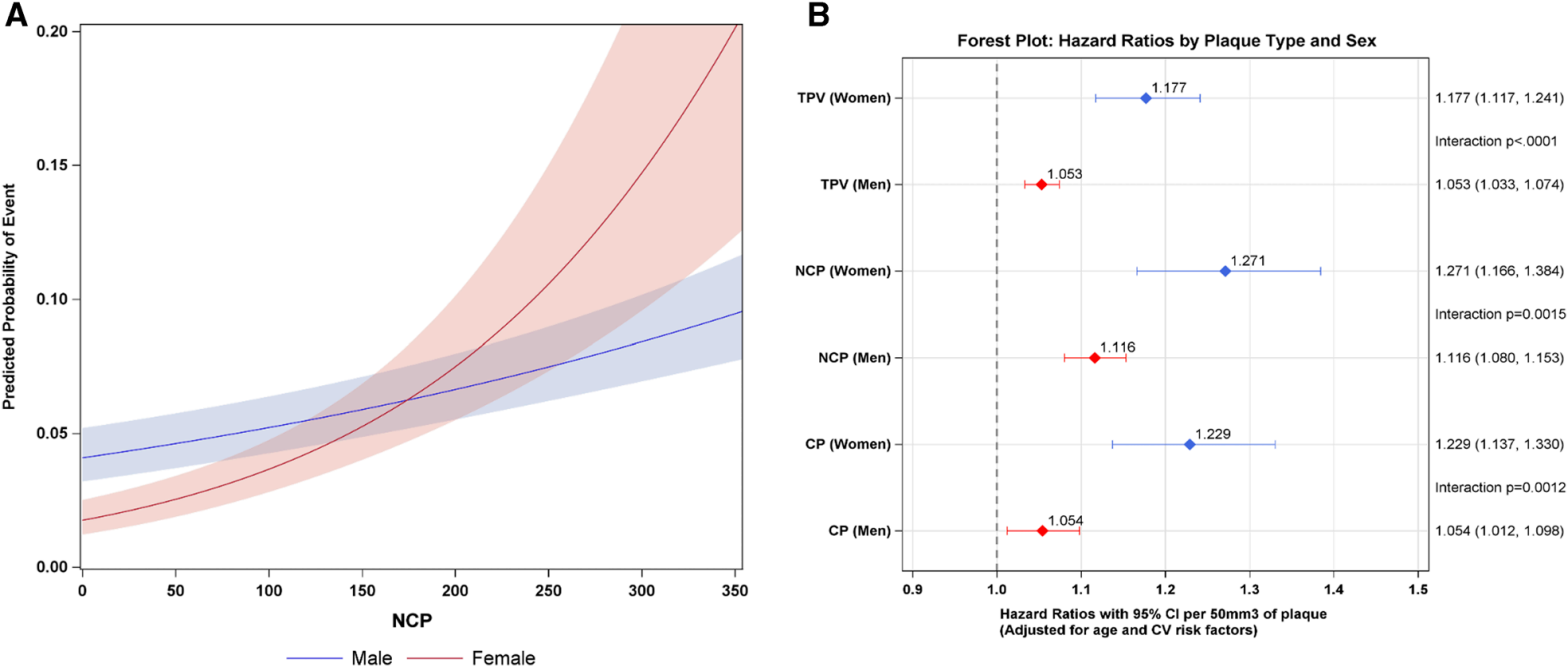
**Predicted probability of major adverse cardiovascular event (MACE) in men vs women and forest plot. A**, Predicted probability by noncalcified plaque (NCP) volume. The probability of MACE increased higher for women compared with men, with an increasing absolute NCP (mm^3^) above a threshold of 180 mm^3^. The outer borders delineate the 95% CIs. **B**, Hazard ratios per 50-mm^3^ increment artificial intelligence–based quantitative computed tomography plaque volume increase for risk of MACE in women vs men (adjusted for age and cardiovascular [CV] risk factors). CP indicates calcified plaque (volume); and TPV, total plaque volume.

Figure 1A shows the stronger association of NCP with MACE in women and exceeding event rates in women for NCP above ≈180 mm^3^.

### Multivariate Analysis

After adjusting for age and CVRF, all AI-QCT features remained independent predictors of MACE in both sexes (Table 4). Similar to the univariate analysis, the plaque burden metrics conferred higher relative risk in women than men (Figure 1B).

**Table 4.**
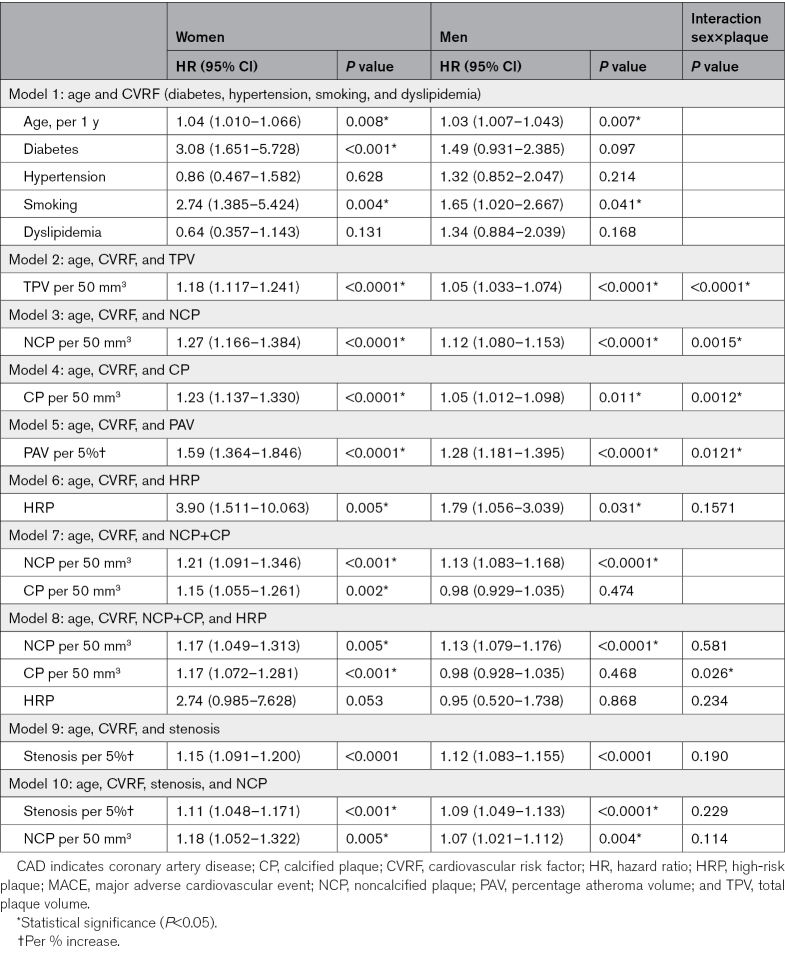
Multivariate Models (Adjusted for Age and CVRF): Prediction of MACE

Specifically, the relative risk increase by 50 mm^3^ of TPV volume was 17.7% (95% CI, 1.12–1.24) in women compared with 5.3% (95% CI, 1.03–1.07) in men (*P*_interaction_<0.001). Similarly, NCP increased the relative risk by 27.1% (95% CI, 1.17–1.38) in women compared with 11.6% (95% CI, 1.08–1.15) in men (*P*_interaction_=0.0015). CP increased the relative risk more pronounced in women by 22.9% (95% CI, 1.14–1.33) compared with only 5.4% (95% CI, 1.01–1.10) in men (*P*_interaction_=0.0012). In contradiction to the plaque burden metrics, DS did not confer heightened relative risk in women versus men (*P*_interaction_=0.1900). When NCP and CP were combined in one model, CP remained predictive in women but not in men. HRP was not significantly associated with MACE when adjusted for NCP and CP in both sexes.

**Figure 2.**
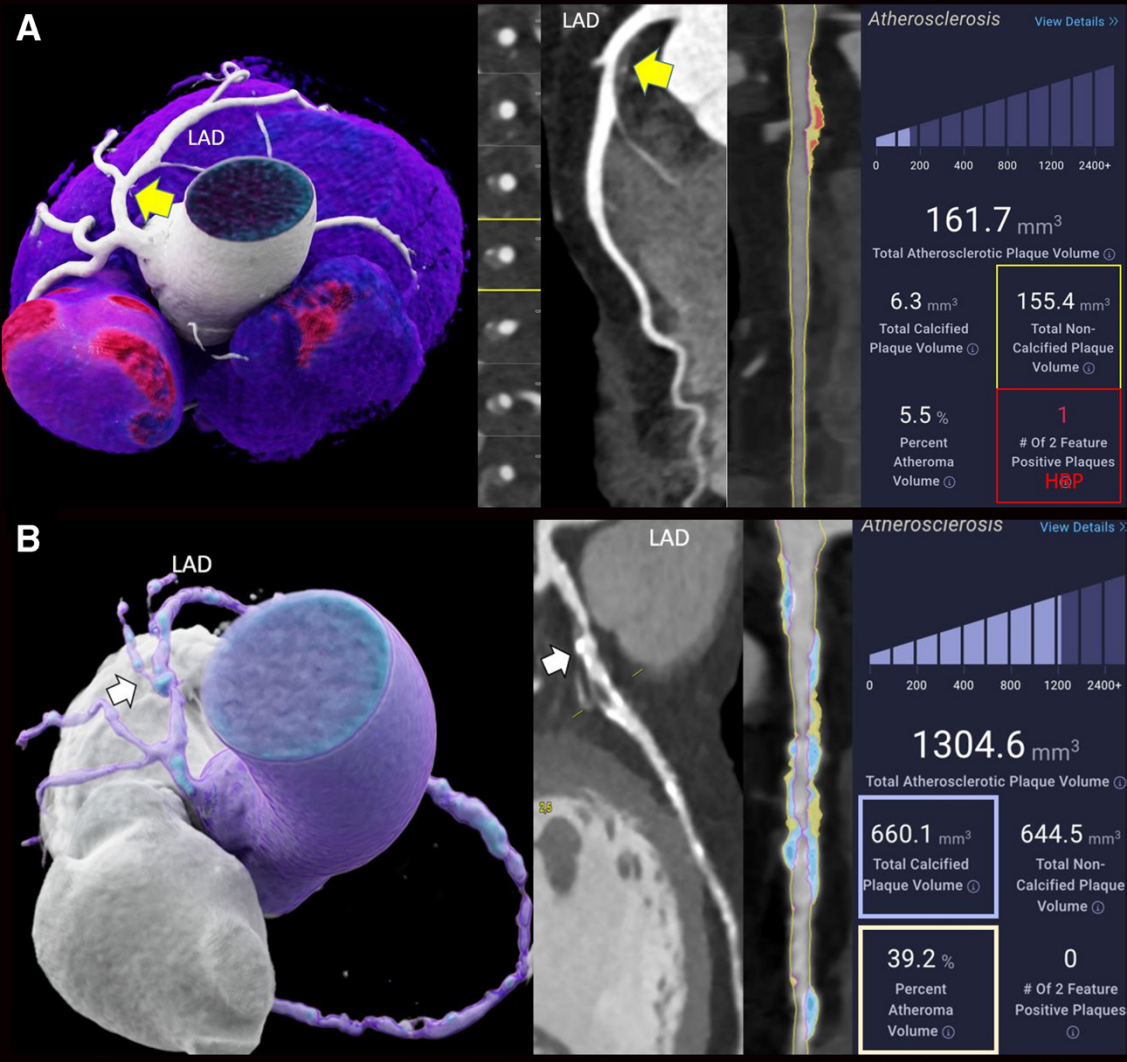
**Case examples. A**, A 55-year-old woman with atypical chest pain and 2 cardiovascular risk factors (CVRFs): arterial hypertension, dyslipidemia, obesity, and hyperuricemia. Coronary computed tomography angiography (CTA) showed a noncalcified plaque (NCP) in the proximal left anterior descending (LAD) coronary artery (yellow arrow) with <50% diameter stenosis. Treatment with lipid-lowering therapy (statin+ezetimibe) and antihypertensive medication was initiated, and she remained event-free. **Right**, Artificial intelligence–based quantitative computed tomography (AI-QCT) plaque analysis showed an NCP volume of 155.4 mm^3^ (yellow box) and high-risk-plaque (HRP; red box) features. Note that color overlay (**mid**) facilitates the delineation of NCP and HRP. **B**, A 65-year-old woman with atypical chest pain, dyspnea, and a high coronary risk profile (5 CVRFs), smoking, arterial hypertension, positive family history, dyslipidemia, diabetes, and high lipoprotein A (89.1 ng/dL), who experienced major adverse cardiovascular event and underwent percutaneous coronary intervention (PCI) (LAD prox/mid/distal and right coronary artery [RCA]). Coronary CTA: white arrow pointing at calcified plaque in the proximal LAD. **Mid**, Curved multiplanar reformation. **Right**, AI-QCT plaque analysis revealed high total plaque volume (stage 3) with high calcified, high noncalcified plaque volume, and high percentage atheroma volume (39.2%).

Figure 2 shows 2 examples of 55-year-old women with NCP and HRP by AI-QCT, in whom medical therapy was initiated and who remained event-free, and a 65-year-old woman with high TPV, high CP, NCP, and PAV who experienced MACE.

### Prognostic Accuracy of Quantitative Coronary Atherosclerotic Plaque Characteristics as Assessed by AI-QCT With MACE

In both women and men, adding TPV, NCP, CP, and PAV to CVRF significantly improved the area under the curve (AUC; Figure S1). From the combined models of CVRF and plaque burden metrics, only for CP, the prognostic accuracy was higher in women than men: AUC, 0.768 (95% CI, 0.71–0.83) versus 0.649 (95% CI, 0.59–0.71; *P*_interaction_=0.0061). Adding HRP to CVRF did not improve the AUC in both women and men. The AUC further improved with the addition of DS. The highest AUC was observed for the 3 combined models CVRF+TPV+HRP+DS in women (c=0.797), CVRF+PAV+DS and CVRF+TPV+DS (c=0.791 for both), and with CVRF+CP+DS achieving a c-value of 0.794 (Table S1).

### DF and AI-QCT Features for Prediction of MACE and Diagnostic Accuracy

Multivariate models adjusted for DF showed similar results for the AI-QCT features than those adjusted for CVRF (Table S2A).

### Receiver Operating Characteristic Curve for Prediction of MACE

DF, combined with AI-QCT features, showed incremental value in both women and men, without sex differences, and adding DS further improved risk stratification (Table S2B). Similarly to receiver operating characteristic curve models combined with CVRF presented in Table S1, the model combining DF+TPV+HRP+ DS (c=0.755) achieved the highest accuracy.

### Secondary End Point (Composite Death and MI)

The rate of death/MI was 1.6% (1.2% for women; 2.1% for men; *P*=0.04). Both univariate and multivariate analyses, adjusted for age and the major CVRF, showed similar results compared with primary end point MACE. The AI-QCT main metrics, TPV, NCP, and CP, were associated with a higher relative risk in women than men (Table S3).

Table S4 shows the multivariate Table 4 including all covariates.

## Discussion

This study, utilizing AI-QCT within the international multicentric Quantitative Coronary Computed Tomography Angiography Evaluation for Evaluation of Clinical Outcomes: an International, Multicenter Registry, highlights the sex-specific differences in the predictive value of coronary plaque phenotypes for MACE. By including symptomatic patients without prior CAD history, this unique cohort provides valuable insights into sex-specific CAD characteristics in the clinically most relevant population. Sex differences in AI-QCT plaque features within such a specific cohort referred to CCTA for clinical indications^9^ have not been previously reported.

Our findings demonstrate that men exhibit a significantly higher total plaque burden, encompassing both NCP and CP, a greater degree and extent of obstructive CAD, and a higher prevalence of HRP features, indicative of a more advanced and aggressive CAD pattern, ultimately resulting in an overall absolute higher MACE rate. These results align with some prior studies^5,29,30^ elaborating on sex differences in plaque burden, composition, and outcomes. However, these studies comprised smaller sample size cohorts or single-center studies, and they did not use AI-QCT.^5,30^ The only study published to date that involved a larger cohort and utilized AI-QCT^29^ did not distinguish between the various plaque components, TPV, NCP, and CP, while the authors only reported PAV. Therefore, our data provide more nuanced and expanded insights into sex-specific differences and their impact on outcomes.

Most strikingly, AI-QCT plaque characteristics independently confer a higher relative risk for MACE in women but not stenosis severity. This is the most relevant and novel finding of the present study, with inherent implications for risk stratification in women.

The pronounced susceptibility of women could be attributed to biological and vascular factors, such as smaller coronary arteries or differences in vasomotor tone and wall thickness, all of which could be influenced by hormonal variations.^2^ Notably, the ability of different AI-QCT features to predict MACE in women varies after adjusting for age and traditional CVRFs; both TPV and NCP remained independent predictors of MACE in both men and women, while CP remained predictive only in women. This discrepancy may be explained by the delayed onset of calcification in women due to menopause and hormonal shifts, and a distinct proteomic signature of CP, defined by 4 proteoglycans, calpoin, protein C, serpin H1, and versican, in women.^31^ This aligns with findings from the Coronary Artery Calcium Consortium data showing that a higher coronary artery calcium score has a more pronounced negative impact on prognosis in women.^7^

Furthermore, NCP volume has emerged as a critical determinant of adverse outcomes in both sexes and might be considered a new biomarker for CAD stability. The ICONIC trial^32^ demonstrated that patients presenting with an ACS had higher baseline volumes of NCP, highlighting its clinical relevance. In contrast, dense CP (≥1000 HU, 1k plaque) rather reflects a stable plaque phenotype with protective effects.^32^ Plaque density can be enhanced with statin therapy, which transforms noncalcified into CP,^33,34^ as shown by the PARADIGM trial^33,35^ and intravascular imaging data.^35^ Thus, NCP burden and, in particular, high-risk features^34^ may serve as a therapeutic target to mitigate plaque vulnerability and improve cardiovascular outcomes.

One potential explanation for the larger relative risk of atherosclerosis in women may be the smaller vessel volume in women and the associated higher coronary plaque burden in women for similar plaque volumes. Interestingly, in our current study, sex differences in PAV^25^ were less pronounced compared with TPV, while the higher relative risk in women persisted, despite PAV already being adjusted for the smaller vessel volume in women. In prior studies, PAV >7% increased MACE risk 3-fold,^11^ and >15.2 PAV indicates a high likelihood of at least 1 vessel with ischemia.^10^

Furthermore, HRP conferred a markedly (5.6-fold) higher relative risk in women compared with men. HRPs are known predictors of MACE,^13,14^ particularly in low-risk patients with low coronary artery calcium scores.^13^ Of note, the overall prevalence of HRP in women was low at 2.5% and lower compared with men, consistent with literature^36^ and the PROMISE trial,^37^ where HRP was shown to be a stronger predictor of MACE in women compared with men (HR, 2.41 versus 1.40).

However, our data suggest that TPV, NCP, and CP may have superior prognostic values compared with the established prognostic value of HRP^13,14^; however, while relative risk was highest for HRP. These results require further investigations in a larger cohort.

Early and accurate diagnosis of nonobstructive CAD is crucial but challenging in the presence of NCP, which are more difficult to detect visually because of their lower contrast with the surrounding perivascular adipose tissue (Figure 2). AI-QCT facilitates NCP detection through automated quantification, improving diagnostic precision, which is especially important in patients with a calcium score zero, in whom the prevalence of NCP ranges between 15.4% and 16%.^38,39^

Finally, AI-QCT consistently outperformed traditional risk scores, such as CVRF and DF models, which exhibited a low accuracy in both men and women. The accuracy of DF was even lower than that of the CVRF in women. When AI-QCT features were combined with conventional risk assessments, accuracy improved and added incremental value for TPV, NCP, CP, and PAV (though not HRP) in both sexes. This trend became more pronounced after incorporating DS.

When considering determinants of outcomes, in men, CAD itself, specifically total plaque burden and NCP volume, emerges as the primary predictor of adverse outcomes, suggesting that anatomic assessment alone may suffice for accurate risk stratification. In women, however, traditional risk factors such as diabetes and smoking history remain independently associated with adverse outcomes, even after accounting for coronary atherosclerosis using AI-QCT. These findings underscore the need to integrate these risk factors into risk assessment, especially for women, not at least because women with type 2 diabetes face a 50% higher risk of fatal coronary heart disease compared with men, as shown in a meta-analysis of 37 studies involving over 447 000 patients.^40^ Another large meta-analysis including over 5 million patients revealed that women with diabetes have a 58% greater risk of coronary heart disease and a 13% higher risk of all-cause mortality compared with their male counterparts.^41^ The reasons underlying these disparities are multifactorial. Women with diabetes often exhibit higher cardiovascular risk profiles, including more severe comorbidities, and are less likely to be prescribed preventive medications such as statins.^3,42^

### Clinical Perspective

The current study shows that symptomatic women with suspected CAD have approximately half the amount of coronary atherosclerosis compared with men and experience ≈50% lower MACE during follow-up. However, the relative risk associated with TPV, NCP, CP, and PAV was higher in women compared with men. As demonstrated in Figure 1A, the higher relative risk relates eventually with higher absolute event rates in women when the NCP exceeds a threshold of 180 mm^3^. These findings may have implications for clinical management including reinforced antiatherosclerotic therapy and preventive interventions. Potentially, women with a higher AI-QCT plaque burden may need more aggressive therapy than men.

Accurate and early detection of NCP burden by AI-QCT is especially important in younger individuals with premature CAD,^43,44^ which face adverse outcomes following an MI (YOUNG-MI trial).^45^ Thus, these features have the potential to improve adverse cardiovascular outcomes in women and enhance the precision of personalized risk stratification. Our study further suggests that AI-QCT features and their sex-specific differences in risk prediction should be included, and treatment and prevention guidelines for CAD^46,47^ may be considered. The 2019 American College of Cardiology/American Heart Association guidelines recommend a high- or moderate-intensity statin regimen for all patients with an elevated atherosclerotic cardiovascular disease risk (≥5%–7.5%) for primary prevention, without distinguishing between men and women.^46,47^ However, these recommendations do not yet incorporate computed tomography angiography findings into their risk assessments.

Advanced AI-QCT features, such as TPV, NCP, and CP volume increase, could be leveraged to guide therapeutic recommendations in both men and women, with a pronounced impact in women. Women exhibiting adverse AI-QCT features may derive additional benefits from reinforced primary preventive measures, including personalized tailored medication regimens such as statin therapy or dose intensification, lower c-LDL (c-low-density lipoprotein) target values, and other interventions such as lifestyle modifications (weight loss, exercise, and nutrition). Statins have been shown to positively affect atherosclerosis by increasing plaque density.^48–50^

Interestingly, while prior studies recruiting patients >1 decade ago (PROMISE trial)^3^ reported a lower statin prescription rate in women, we found no difference in statin intake in our more contemporary cohort, enrolling patients who underwent CCTA within the last 5 to 7 years. This suggests a positive trend, possibly driven by public health campaigns such as the American Heart Association-Go Red for Women initiative.

Finally, emerging technologies such as photon-counting detector computed tomography offer the potential to further enhance the accuracy of AI-QCT through enhanced spatial resolution.^51^ These advancements could further improve the precision of personalized cardiovascular risk stratification.

### Study Limitations

The retrospective study design with inherent biases must be acknowledged as a study limitation. Medication was only collected at baseline, and any changes during the follow-up period were not recorded. The event rate of the secondary end point (death/MI) was low; therefore, too definite conclusions cannot be drawn yet.

### Conclusions

Despite the total AI-QCT CAD burden being higher in men, similar increments in AI-QCT-derived plaque features (TPV, NCP, CP, and PAV) confer a significantly higher relative risk for MACE women versus men and added incremental value to traditional cardiovascular risk assessment. Therefore, integrating quantitative AI-QCT parameters offers a more accurate risk estimate in women. Using AI-QCT CAD feature-based risk stratification for MACE instead of relying on traditional risk scores has the potential to enhance the precision of cardiovascular risk stratification in women.

#### Clinical Relevance

AI-QCT is especially suited to women and should prompt increased awareness of higher risk, which might be followed by more aggressive antiatherosclerotic therapy and reinforced preventive interventions.

## Article Information

### Acknowledgments

Principal investigators (PI): Alexander van Roesendael and Ibrahim Danad. The PI (Drs Danad and van Rosendael) had full access to all the data in the study and takes responsibility for the integrity of the data and the accuracy of the data analysis.

### Sources of Funding

The study is sponsored by Cleerly, Inc. The authors are solely responsible for the design, methodology, and conduct of the study, statistical analysis, drafting of manuscripts, and the final publications.

### Disclosures

The authors declare the following financial interests/personal relationships that may be considered potential competing interests. Dr van Rosendael is a member of the Cleerly Scientific Advisory Board. Dr Pontone received an honorarium as a speaker/consultant and an institutional research grant from GE Healthcare, Bracco, Medtronic, and Novartis. Dr Buechel reports receiving speaking honoraria from GE Healthcare, Pfizer, Gilead, and IBA. Dr Gräni received funding from the Swiss National Science Foundation, InnoSuisse, the CAIM Foundation, the GAMBIT Foundation, and the Novartis Foundation for biomedical research, outside of the submitted work. Dr Choi is a consultant for Siemens, holds equity in Cleerly, and receives grant support from the George Washington Heart and Vascular Institute. Dr Rochitte reports receiving speaking honoraria for Pfizer, Edwards, GE, and Manole. Dr Khalique is a consultant for Edwards, Croivalve, and Restore Medical, holds equity in Triflo, and received honoraria for educational programs from Heartflow. Udo Hoffmann is an employee and equity holder in Cleerly, Inc, and received honoraria from Stanford University, Clinical Cardiovascular Sciences, Rapid AI, and MedTrace. Dr Danad is a member of the Cleerly Scientific Advisory Board. Dr Marques is a consultant for Cleerly, Inc. The other authors report no conflicts.

### Supplemental Material

Tables S1–S4

Figure S1
